# Effect of Multi-Source Ultrasonic on Segregation of Cu Elements in Large Al–Cu Alloy Cast Ingot

**DOI:** 10.3390/ma12172828

**Published:** 2019-09-03

**Authors:** Hao Peng, Ruiqing Li, Xiaoqian Li, Shan Ding, Mengjun Chang, Liqing Liao, Yun Zhang, Pinghu Chen

**Affiliations:** 1 College of Mechanical and Electrical Engineering, Central South University, Changsha 410083, China; 2 Light Alloy Research Institute, State Key Laboratory of High Performance Complex Manufacturing, Central South University, Changsha 410012, China; 3 School of Automation, Central South University, Changsha 410012, China; 4 School of Advanced Materials, Peking University Shenzhen Graduate School, Shenzhen 518055, China

**Keywords:** large-scale, Al–Cu alloy, segregation, solidification, ultrasonic

## Abstract

The structure and composition of large-scale Al–Cu alloy ingots are inhomogeneous, and the segregation of (especially) elemental Cu negatively affects the uniformity and stability of the subsequent components. In this work, four ultrasonic generators were used to manipulate solidification/microstructures of cylindrical Al–Cu ingots (1250 mm in diameter; 3500 mm in length). The influence of ultrasonic configuration on both solidification microstructures and solute macrosegregation was investigated by changing the position parameters of generators for a fixed power. The results revealed that when the ultrasound is applied close to the center (I) from the 1/2 radius (II), the grain structure of the center undergoes significant refinement, degree of positive segregation in the center can be reduced, segregation index decreased from 0.2 to 0.15, and range of positive segregation in the center decreased from 200 to 150 mm. The segregation of elemental Cu was weakened by the combined effects of the ultrasound on the flow, heat transfer, and grain movement.

## 1. Introduction

Al–Cu alloys are widely used in aerospace structural parts. At present, with the development of aerospace structural parts for integration and large-scale applications, the size of Al–Cu structural parts is increasing. The specifications and quality requirements of Al–Cu alloy ingots for manufacturing components are also increasing. However, the manufacturing process of large-scale ingots is difficult, owing to the complexity and solidification of the alloy composition characterizing the billets. The inhomogeneity of the temperature field results in an uneven distribution of the ingot structure and composition. The differences of the structure, alloy elements, and crystalline-phase state are especially stark between the core and epidermis of the ingot. This inhomogeneity has considerable influence on the follow-up performance.

Currently, the main measures used to improve the casting quality are optimization of casting process conditions; addition of refiners and modifiers; and dynamic refinement (such as mechanical stirring [1,2], electromagnetic stirring [3], ultrasonic vibration [4,5], and bubble stirring [6]). Among these measures, high-quality ingots can be obtained by optimizing the casting process. This method has limited effect, however, on improving the casting structure and extremely homogenous high-quality ingots are unattainable. Although the modification method can effectively refine the grain structure, segregation precipitation occurs easily for large particle sizes. Furthermore, the complexity of operation is increased because of the inhomogeneity of the ingot structure and the choice of refiner for different target alloys. In contrast, the method of the external physical field has the advantages of good refining and no pollution or damage to the alloy. For example, the ultrasonic directly applied to any part of the mold can directly act and refine the central structure of the ingot.

A typical defect that occurs in a wide range of casting processes, i.e., macrosegregation, refers to an inhomogeneous distribution of alloying elements in the casting products. Based on extensive simulation and experimental investigation, mechanisms/theories of macrosegregation have been proposed. These include mechanisms based on thermosolutal convection [7], transportation of solid grains in the slurry and upper mushy zone [8,9,10], and shrinkage-induced flow and deformation of the solid network in the mushy zone [11]. The natural thermosolutal convection in direct chill (DC)-cast Al alloy ingots enhances the positive centerline macrosegregation, but the shrinkage-induced flow transports the solute-enriched liquid away from the central part of the castings, resulting in negative centerline segregation. In large-scale castings, duplex structures, including coarse and fine dendrites, have been frequently observed in the central part of DC-cast ingots. Moreover, some grains can float in the slurry zone and then settle down [12,13,14]. Controversies persist in the modern theories of macrosegregation, where the contribution of free-floating grains to the observed chemical inhomogeneity remains debatable [13]. A single mechanism has never fully explained all the experimental results. Actually, macrosegregation results from the consequences of the intricate interaction between multiple mechanisms [15,16,17]. A commonly observed phenomenon, surface-to-surface distribution of alloying elements (in a cross-section of a DC-cast ingot), implies distinct regions of positive (solute-enriched) and negative (solute-depleted) segregation [18]. This yields a significant decrease in the solute near the centerline, adjoined by a positive segregation zone spreading into the outward direction, an adjacent thin negative segregation zone, and another positive segregation layer at the surface [19].

In terms of large-scale Al alloy ingots, a defect, such as macrosegregation, is quite complex due to the combined effect of large-scale solidification volume, non-uniform temperature field, and solidification microstructures. Macrosegregation in large-scale ingots with diameters of 1200 mm has yet to be explored via either experiment or simulation.

Therefore, in this work, four ultrasonic generators were used to manipulate the solidification/microstructures of cylindrical Al–Cu ingots (1250 mm in diameter and 3500 mm in length). The influence of ultrasonic configuration on both the solidification microstructures and the solute macrosegregation under the same power was investigated by varying the position parameters of the generators.

## 2. Experimental Procedure

### 2.1. Materials and Process

**Figure 1:**
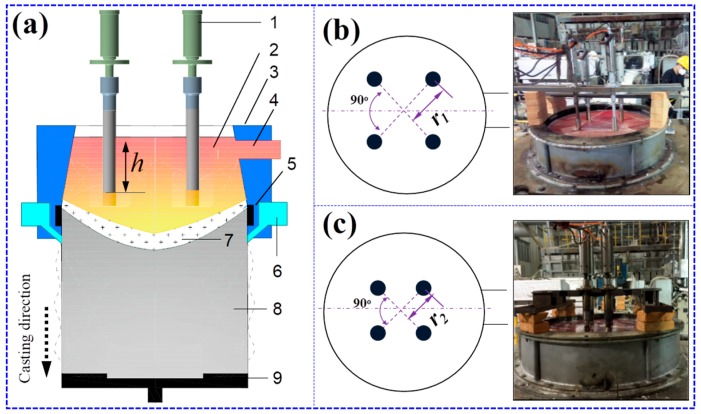
(**a**) Schematic showing configuration of the multiple ultrasonic-generators in the direct chill (DC) large-scale castings: 1—ultrasonic generator, 2—melt, 3—mold with a hot top, 4—launder, 5—crystallizer, 6—cooling system, 7—mushy zone, 8—solidfied ingot, 9—dummy plate. (**b**,**c**) Schematic and photograph of I and II.

An Al–Cu alloy (chemical composition: Al–6.2Cu–0.36Mn–0.11Zr–0.1V–0.10Fe–0.06Si–0.01Mg–0.10Zn–0.05Ti (wt.%)) was investigated in this work. Unless specified otherwise, all element contents are expressed in weight percent. The Al–Cu alloys were melted in an electrical furnace, and were then subjected to stirring, degassing, decontamination, filtering, and purification. The entire melting process was performed under a protective argon atmosphere. Combined with spinning nozzle inert gas flotation (SNIF®), the fumeless inline degassing (FILD®) method was used to decontaminate the alloy melts. The melts were then introduced into the casting system. During solidification, the cooling water was sprayed on the ingot surface until the casting was completely solidified. Four ultrasonic generators were employed with the same frequency, power, and peak-to-peak amplitude of 20 ± 1 kHz, 1 kW, and 20 ± 1.0 μm, respectively. A schematic showing the configuration of the multiple ultrasonic generators in the direct chill (DC) casting is presented in Figure 1. The values of h, r_1_, and r_2_ are 300, 300, and 200 mm, respectively. Ultra-large 2219 Al alloy ingots with a diameter of 1250 mm and a length of 3500 mm were manufactured by ultrasonic treatment technology.

### 2.2. Methods

**Figure 2:**
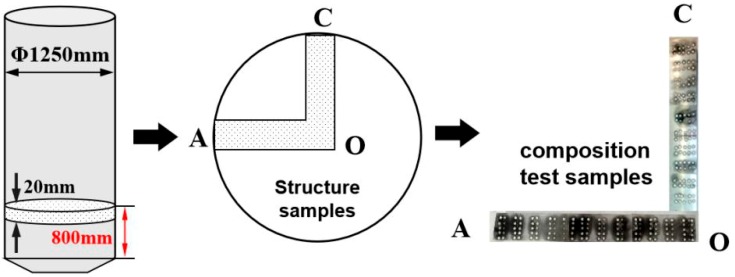
Schematic showing the sampling positions for microstructural and chemical analysis of the large-scale 2219 Al alloy ingot.

After the homogeneous annealing treatment, two cross-sections (each with a thickness of 20 mm) from each of two ingots were selected for study at the ultrasonic-vibration steady stage of the ingots as showed in Figure 2. The samples of micro- and macrostructures were cut at different locations along the billet radius. Subsequently, the metallographic samples were mechanically ground and polished, followed by etching with Keller’s solution (1% HF, 1.5% HCl, and 2.5 vol.% HNO_3_). The grain morphology of these metallographic samples was examined using a DSX50240 optical microscope (OM) (OLYMPUS (China) Investment Co., Ltd., Beijing, China) equipped with image analysis software. Average grain sizes (d) measured by a linear intercept technique (ASTM 112-10), and the cross-section comprising each billet were used to assess the refining efficiency. Moreover, the distribution of main solute (Cu) in each sample was measured by a spark spectrum analyzer (SPECTROMAXx, Spectro Analytical Instruments, Germany).

Macrosegregation or the lack thereof can be quantified by determining the distribution of Cu content across the ingot. The index, Δ*C*, which is used to evaluate the macrosegregation degree of Cu, is determined from:

(1) $$\Delta C=\frac{C_{i}-C_{o}}{C_{o}}$$

where *C_i_* and *C_o_* are the Cu content at a tested location and average Cu content of the ingot, respectively. Δ*C* indicates the degree of deviation from the average value of the copper element in this region.

The macrosegregation ratio (*S*) is defined as follows [3,6]:

(2) $$S=\Delta C_{\max}-\Delta C_{\min}$$

*S* was used to evaluate the fluctuation range of macrosegregation across the entire cross-section of the ultra-large ingots. That is, *S* increased with an increasing macrosegregation degree of the element in the ingot, and unevenness of the solute distribution.

Δ*r* was used to evaluate the fluctuation range of the grain size, and was determined from:

(3) $$\Delta r=r_{\max}-r_{\min}$$

where *r*, which is the grain size of a certain tested location, indicates the uniformity of the grain.

## 3. Experimental Results

### 3.1. Macrostructure

**Figure 3:**
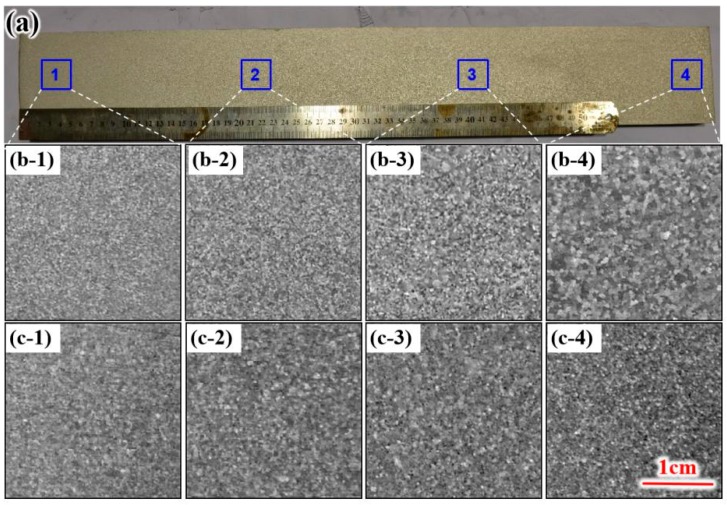
Macrostructure of the two ingots. (**a**) Macrospecimen, (**b**) ingot I, (**c**) ingot II.

Figure 3 shows the macrostructure of the two casting ingots; (a) shows the macroscopic test, (b) and (c) shows the macrostructure occurring in the same locations in the cross-sections of the two casting ingots. Regions lying along the traverse from the ingot edge to the heart are shown in panels 1 to 4. From the illustration, we know that, compared with those of ultrasonic ingot II, the edge structure, heart structure, and core structure of ultrasonic ingot I are finer, thicker, and coarser, respectively.

### 3.2. Microstructure

**Figure 4:**
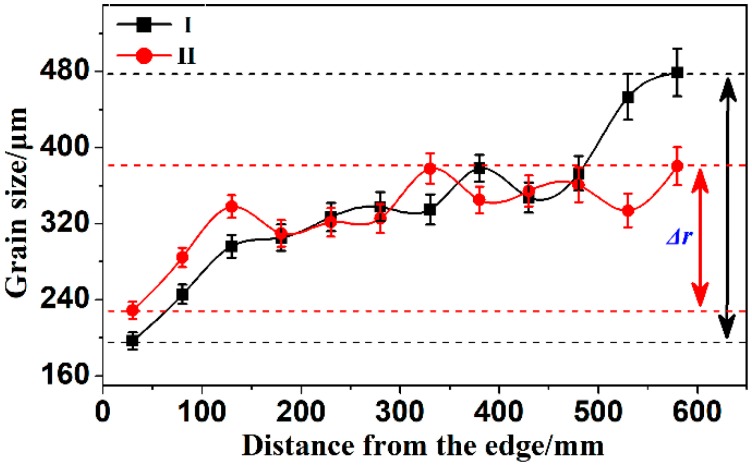
Grain size distribution plots of the two ingot cross-sections.

Figure 4 shows the grain size distribution schematic of two ingot cross-sections, which are from the edge to the center along the radius. The largest deviation of the two casting ingot grain sizes is shown by two vertical arrows. From the schematic we know that:

(1) The grain size gradually increases within 0 to 150 mm from the ingot skin, and the grain size is stable from 150 to 475 mm. Then, the grain size of group I increases sharply from 475 mm to the ingot heart.
(2) The casting grain size of group I was smaller than group II universally, which was in the fine-grained area of the edge. However, the casting ingot in group I was significantly higher than the grain size in group II when in the heart location.
(3) The maximum deviation of the grain size in group I was 282 μm (Δ*r*), which was higher than the same size in group II. The value of the ultrasonically untreated ingot is 300 μm [20].

**Figure 5:**
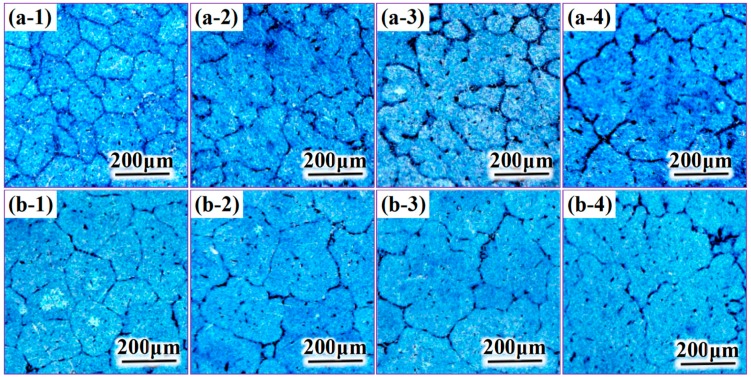
Comparison of microstructure at the same position on the cross-sections of the two ingot types. (**a**) Ingot I, (**b**) ingot II.

The microstructures occurring in the same place of the two casting ingot cross-sections are compared in Figure 5. Ingot I is shown in (a) and ingot II is shown in (b). Moving from the ingot edge to the heart, the regions are denoted as 1 to 4. The grains in the edge of ingot I are equiaxed and finer than those in other locations. Moreover, the grain size increases toward the ingot center, and the morphology remains equiaxial or approximately equiaxial. However, the microstructure of the ingot core is characterized by petal-shaped grains with coarse dendrite arms. The results of the microstructure comparison can be summarized as follows: Considerable grain size refinement occurs in the ingot edge and the ingot heart of group I and group II, respectively.

**Figure 6:**
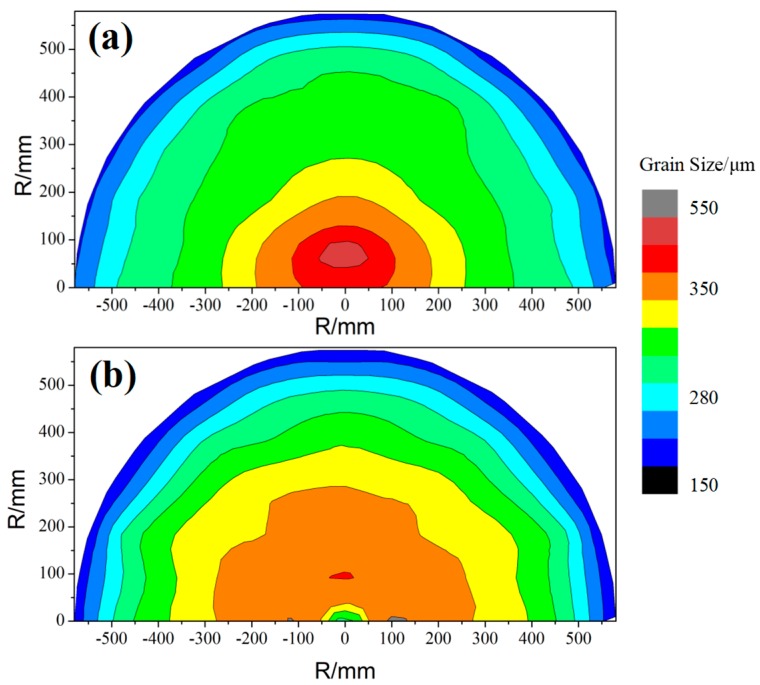
Comparison of the microstructures comprising the cross-sections of the two ingots. (**a**) Ingot I, (**b**) ingot II.

The grain size distribution nephograms obtained for the cross-section of the two ingot semicircles are compared in Figure 6. As the figure shows, the structures of solidification differ significantly among the three areas, i.e., surface, middle, and center. Comparing group I and group II reveals that: (1) The area of the epidermal and cardiac grains of group I is smaller than that associated with group II, and the middle region is larger; and (2) the heart size of the coarse grains in group II and the area of the middle region decrease. The results reveal that the ultrasound has a greater effect on the refinement of the cardiac part of group II (than on group I), and the refining effect of the solidified tissue in the middle region is weak.

### 3.3. Macrosegregation of Elemental Cu

**Figure 7:**
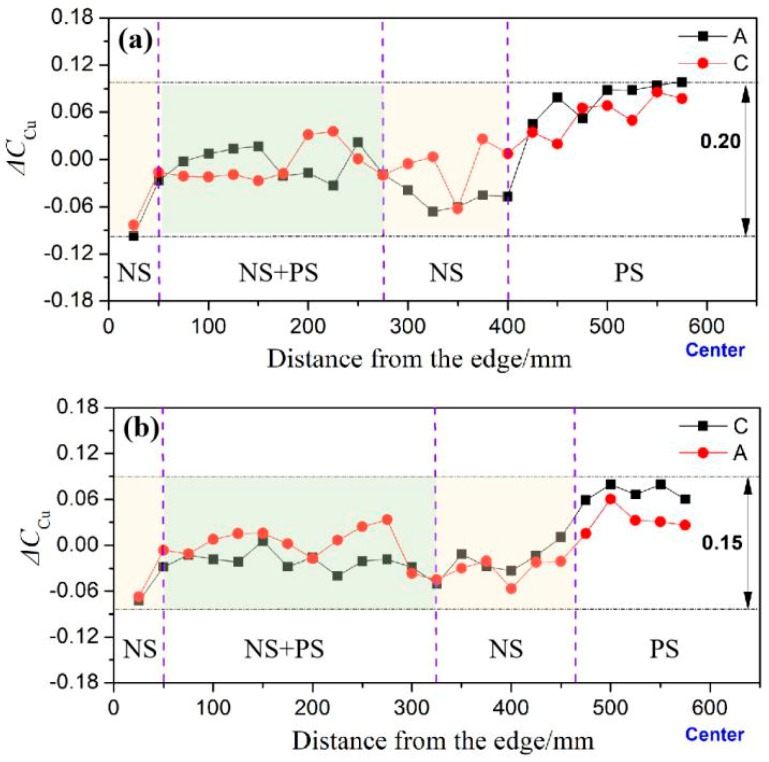
Segregation rate distribution of Cu element on the cross-section of the two ingots. (**a**) Casting ingot I, (**b**) casting ingot II.

The distribution curve of the segregation rate is shown in Figure 7. In the plot, the segregation rate of elemental Cu is represented by the ordinate, and the distance from the check point to the ingot edge is represented by the abscissa. The segregation rate of Cu, in the ultrasonic ingot A, C board of group I and the segregation rate in the ultrasonic ingot A, C board of group II are shown in Figure 7a and b, respectively. “NS” stands for negative segregation and “PS” stands for positive segregation in the figure. A comparison of the two schematics reveals that the segregation of ingots in the two groups proceeds from the edge to the center: Negative segregation (edge of the ingot, “NS” in Figure 7) → stationary phase (“NS + PS” in Figure 7) → negative segregation (“NS” in Figure 7) → positive segregation (heart of the ingot, “PS” in Figure 7). Nevertheless, significant differences between the segregation of the ingots are observed and summarized as follows:

(1) The degree and area of positive segregation in the heart of casting ingot II decrease (maximum value of positive segregation is 0.08, range is 150 mm). However, the maximum value and range of positive segregation in the heart of the casting ingot I are 0.1 and 200 mm, respectively.
(2) The range of negative segregation in the middle portion of casting ingot II increases, and the area of negative segregation moves gradually toward the heart integrally. To be specific, the range of negative segregation area of casting ingot I is 125 mm, whereas the corresponding value for group II is 150 mm.
(3) The degree of overall segregation decreases in casting ingot II. Similarly, the segregation index of casting ingot I is 0.2 (S), whereas the segregation index of casting ingot II is 0.15. For the ingot without ultrasonic treatment, the concentration of Cu fluctuated in a large range (S = 0.23), varying from 0.10 at the edge to 0.13 at the central positions [21].
(4) The scope of the stabilization phase of casting group I is 225 mm, and the scope of group II is 275 mm.

The above experimental results reveal that when ultrasonic action occurs close to the center (ultrasonic ingot II) from the 1/2 radius (ultrasonic ingot I), the grain structure in the center ingot undergoes significant refinement. Furthermore, the degree and area of center positive segregation decrease, area of negative segregation in the middle section moves to the ingot center, and the range of the steady phase increases.

## 4. Discussion and Analysis

### 4.1. Influence of Ultrasonic Action on Macrosegregation of Large-Scale Ingot

**Figure 8:**
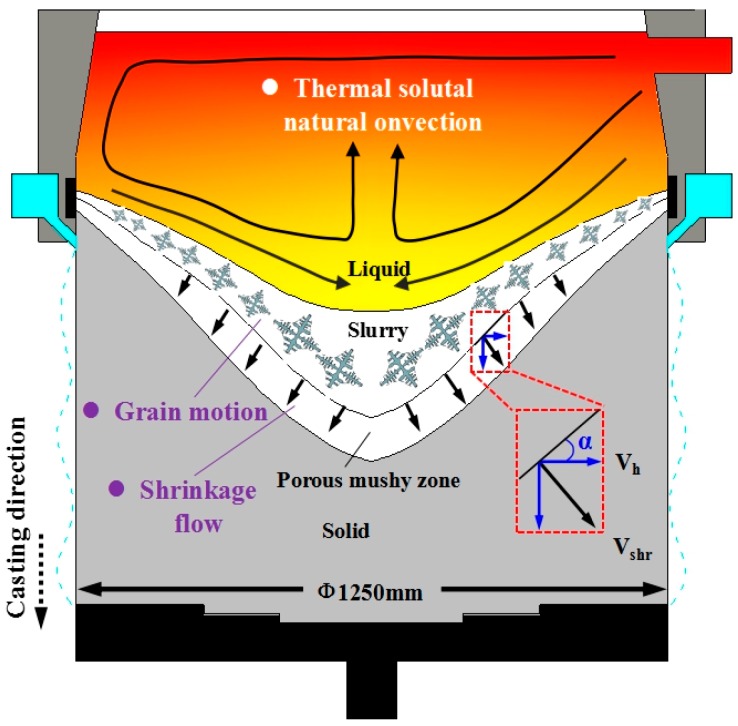
Schematic showing macrosegregation mechanism in a large-scale ingot.

Figure 8 shows the formation mechanism leading to composition macrosegregation during semi-continuous casting. From top to bottom, the ingot is divided into a liquid phase zone, solid–liquid coexistence zone, and solid phase zone. The coexistence zone is divided into the slurry zone and mushy zone [19].

The solute distribution in the liquid phase zone is affected mainly by natural convection (also referred to as thermosolutal convection) resulting from temperature and solubility differences.

The solid grain can move freely in the slurry zone. The solute distribution is affected mainly by the free movement of solid grains with a relatively low solute content. Dendrites overlap in the mushy zone, forming a solid skeleton, and the grains are unable to move freely. The solute distribution is then affected by the shrinkage-induced flow and deformation of the solid network.

#### 4.1.1. Effect of Ultrasonic Action on Segregation Caused by Thermosolutal Flow

Consider the semi-continuous casting of an aluminum alloy. The difference in the density of the aluminum liquid results from the difference in the density of the liquid in different positions. This difference is induced by the difference in the temperature of the first solidification region and the internal post-solidification region. Furthermore, the non-uniform distribution of the solute elements in the alloy during semi-continuous casting results in relative movement between the liquid. The fine movement of each part is accumulated and superposed and hence, convection is generated in the molten pool. The melt with the higher solute concentration (than the rest of the molten pool) is gradually pushed to the bottom of the pool along the solidification front edge. Therefore, the core part of the cast ingot undergoes positive segregation, and the side part undergoes negative segregation. The methods for changing the flow field in the semi-continuous casting pool will change the state of the segregation.

The thermosolutal flow increases (in general) the macrosegregation in the billet center, especially when instability leads to the emergence of relatively small flow cells. These cells result in improved transport of solute-rich liquid from the mushy zone. The flow is generally intensified by a relatively deep liquid pool and high casting temperatures.

Thermal–solutal convection is initiated due to the temperature and concentration gradients and the corresponding buoyancy term in the momentum equation is related to the temperature and average concentration by [22]:

(4) $$F=-\rho_{l}\left[\beta_{T,l}\left(T-T_{o}\right)+\sum_{i=1}^{n}\beta_{C,l}^{i}\left(C_{l}^{i}-C_{o}^{i}\right)\right]g,$$

where *β*_*T*,*l*_ and *β*_*C*,*l*_ are the thermal and solutal expansion coefficients of the liquid phase, and *ρ_l_*, *T*, *C_l_*, *T_o_*, *C_o_*, *g*, and *i* are the liquid density temperature, liquid concentration, reference temperature, reference concentration, gravity acceleration, and index related to the solute elements, respectively. The aforementioned equation indicates that buoyancy can be reduced by a uniform distribution of temperature and composition in the molten pool.

The introduction of an ultrasound into the melt can yield increased uniformity of the temperature distribution in the bath [5]. This leads to a reduction in the temperature difference (*T* − *T_o_*) in Equation (4) and the degree of macroscopic segregation induced by temperature gradients.

#### 4.1.2. Effect of Ultrasonic Action on Segregation Caused by Grain Movement

The movement of free crystals is one of the main causes of negative segregation in the center of large ingots. Due to gravity, solute-poor solid grains move along the solidification front to the bottom of the liquid cavity, thereby leading to negative segregation in the central region. The influence of ultrasonic action on grain growth and nucleation has been analyzed. The high temperature and high pressure produced by ultrasonic cavitation can promote nucleation on the one hand, and on the other hand, increase the wettability of heterogeneous particles. This increased wettability leads to an increase in the number of nucleation cores of the grains. The stirring and scouring effects caused by the ultrasonic sound flow yield a uniform distribution of these cores and scouring of the primary dendrites at the solidification front. The resulting increase in the number of free grains, and the refinement of grains will reduce the degree of positive segregation in the center.

Moreover, the density of the solid phase is higher than that of the liquid phase. Therefore, the flow in the slurry zone corresponds to the settling of suspended solid grains during their movement along with the liquid. Ni and Incropera [23] suggested that this flow can be described by:

(5) $${\vec{V}}_{S}-{\vec{V}}_{l}=\frac{1-g_{s}}{18\mu_{m}}\left(\rho_{s}-\rho_{l}\right)d_{s}^{2}g,$$

where ${\vec{V}}_{S}$ and ${\vec{V}}_{l}$ are the velocities of the solid and liquid phases, respectively; *g_s_* is the fraction of the solid; *ρ_s_* is the solid density; *d_s_* is the grain size; *g* is the gravity acceleration, and *μ_m_* is the apparent viscosity of the semi-solid mixture.

The size of the floating grain in the liquid phase lies between the critical nucleus radius and the grain size of the final ingot. Results of the ultrasonic casting experiment reveal that the grain size of the ingot decreases under the action of an ultrasonic external field. According to Equation (5), for decreasing, the relative motion of liquid and solid can be weakened, and the segregation degree of the solute elements can then be reduced.

#### 4.1.3. Effect of Ultrasonic Action on Segregation Caused by Solidification Shrinkage

The shrinkage-induced flow is directed normal to the solid (or coherency) fraction contour, toward the relatively deep part of the mushy zone. This flow contributes to the negative centerline segregation and positive surface segregation [9,24].

Based on these considerations, the proposed model links the macrosegregation to the local slope of the coherency isotherm (*a*), thickness of the mushy zone (*L_m_*), shrinkage ratio (*b*), and solidification path of an alloy (accounted for in the coefficient, *A*) through a so-called horizontal solute transfer distance [11]:

(6) $$L_{h}=0.78C_{o}L_{m}\beta \left(\sin2\alpha \right)/2,$$

where *L_m_*, *β*, and *α* are the vertical dimension of the mushy zone, volumetric shrinkage (can be taken as 0.1 for aluminum alloys), and the angle between the tangent to the coherency isotherm and the horizon, respectively. The derivative of Equation (6) with respect to the radial distance from the billet center (*R*) will lead to the net efflux and is a measure of the macrosegregation caused by solidification shrinkage; (*dL_h_/dR*)/*C_o_* reflects the relative segregation. The above formula can be used to roughly estimate the degree of shrinkage-induced macrosegregation.

**Figure 9:**
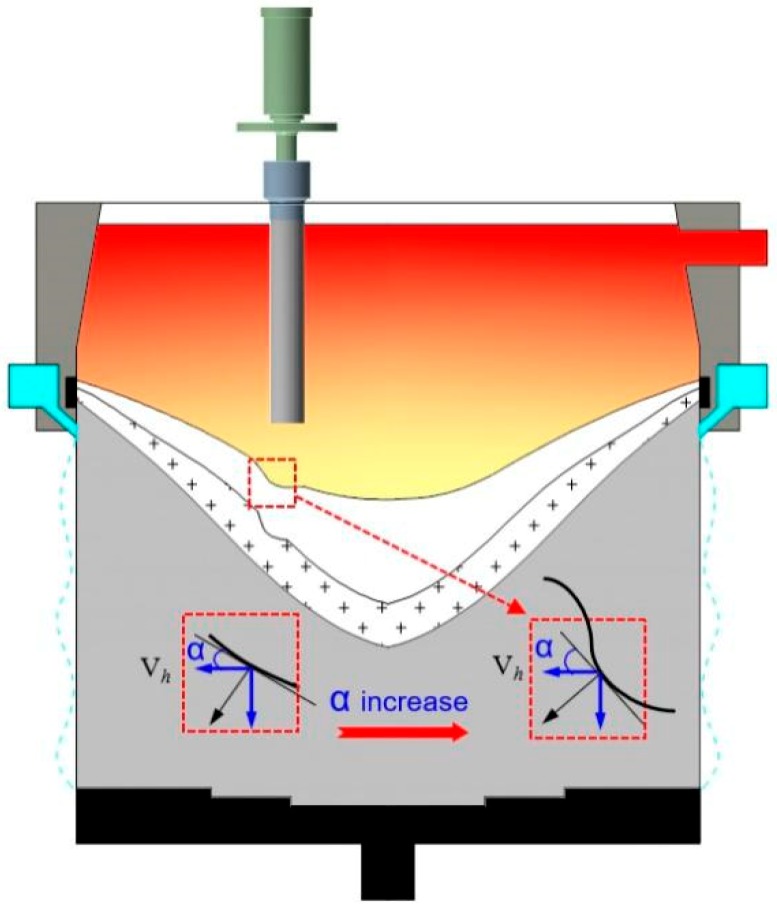
Effect of ultrasonic changes on the solidification front.

According to previous studies [5,25], the initial freezing shell point moves down, the billet sump becomes shallow and flattened, i.e., the shape of the solidification front becomes shallow, and *L_m_* as well as the angle, *α*, decrease when the ultrasonic application is reasonably used. According to Equation (6), the negative segregation caused by solidification shrinkage will weaken with the decrease of *L_h_*. However, if the ultrasonic radiation rod is close to the solidification front, the impact produced by the ultrasonic will lead to a concave of the local solidification front. In the present work, this corresponding thickening solidification front has an effective effect on the increase of the negative segregation degree of *L_h_*, as shown in Figure 9.

The macro segregation is a complex result of multi-factor interaction. The influence of ultrasonic on this segregation depends on the mechanism of the occurrence of segregation. Consequently, ingots with different macro-segregation states are obtained with the complex ultrasonic effect.

### 4.2. Effect of Different Ultrasonic Horizontal Position on Segregation in a Large Al–Cu Alloy Ingot

**Figure 10:**
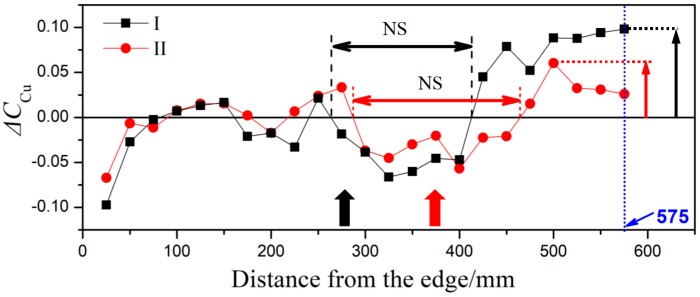
Segregation curves corresponding to the two groups of ultrasonic ingots with a diameter of Φ1250 mm.

The segregation curves of ingot I and ingot II were further analyzed, as shown in Figure 10. The radiation rod lies 300 mm from the ingot center, as indicated by the black solid arrow in group I of Figure 10. Additionally, the red solid arrow in group II represents that the rod lies 200 mm from the center. A comparison analysis of the two curves was made and the result reveals that:

(1) Area of ultrasonic action: When the ultrasound rod is moved from edge to the center, significant negative segregation (see figure) occurs in ultrasound group I (denoted by squares representation), which lies 185 to 330 mm away from the center. The same trend is observed for ultrasound group II (denoted by circles). However, the range of the negative segregation region is 130–310 mm. Additionally, the ultrasonic has a positive effect on the occurrence of negative segregation in the local region.
(2) Area of the ingot center: The degree of positive segregation in group II is reduced, as marked by the right-most arrow in the figure, which indicates that the ultrasound rod is approaching the center of the ingot. Therefore, the negative segregation has an increased tendency in the local region and that contrasts the positive segregation.

**Figure 11:**
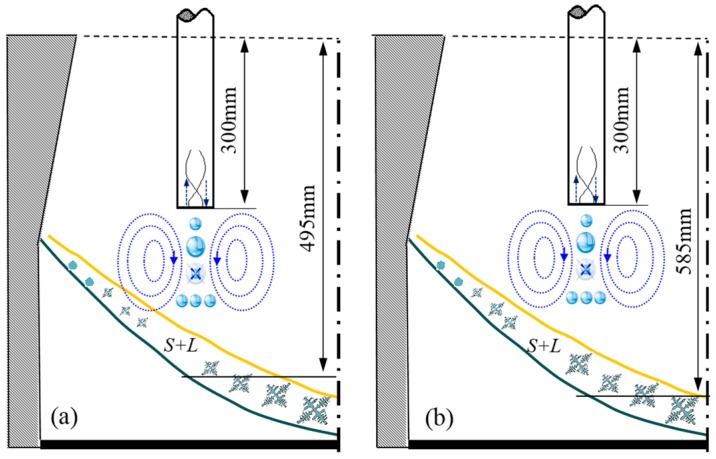
Ultrasonic mechanism of two Φ1250-mm groups. (**a**) Casting ingot I, (**b**) casting ingot II.

The mechanism of these two different ultrasonic-casting groups is shown in Figure 11. The ultrasound rod is placed respectively at the position one-half of the radius and one third of the radius of the ingot, as shown in (a) and (b). The maximum depth of the core melt level is 790 mm in the 1250-mm hot-top semi-continuous casting system. Besides, the effective depth of the ultrasonic effect in (a) and (b) is 495 and 585 mm, respectively. The liquid cavity is steep. Although the cavitation area under the radiation rod is limited by the ultrasonic, the solid–liquid coexistence area is still influenced by the microjet formed via cavitation and other impact action. The number of primary crystal cores in the ultrasonic area increases owing to the scour effect of the microjet (as seen in Figure 11). The corresponding stir effect of the ultrasonic flow promotes the slip of free grains and this movement ultimately leads to negative segregation. In addition, by pushing down the solidification front, the microjet impact at the end position of the ultrasonic radiation rod has a certain influence on the liquid cavity. The aforementioned analysis indicates that ultrasound-induced changes in the shape of the liquid cavity lead to an increase in the angle (α) between the solid–liquid boundary and the horizontal line. Consequently, solidification shrinkage occurs with the increase of the degree of negative segregation.

In conclusion, the stir effect of the ultrasonic can effectively increase the number of free grains, and obviously promote the movement of these grains, which leads to the occurrence of negative segregation. On the other hand, the downward impact action generated by the radiation rod leads to an increase in the angle (α) between the solid–liquid boundary and the horizontal line. Moreover, the degree of negative segregation increases with the increase of the inverse flow, leading to the occurrence of solidification shrinkage.

## 5. Conclusions

Two cylindrical Al–Cu ingots, with a diameter of 1250 mm, were manufactured by an ultrasonic-assisted casting system with different position parameters of the ultrasonic generators. The influence of ultrasonic configuration on both the solidification microstructures and the solute macrosegregation was investigated under the same power of the generator. The results revealed that when the ultrasound is applied close to the center (II) from the 1/2 radius (I), the grain in the center of ingot undergoes a significant refinement. Furthermore, the degree of positive segregation in the center obviously reduced, the segregation index decreased from 0.2 to 0.15, and the radius of positive segregation in the center decreased from 200 to 150 mm. The segregation of the Cu element is weakened by the combined effects of the ultrasound on the melt flow, heat transfer, and grain movement.
